# Marine mammal acoustic detections in the Greenland and Barents Sea, 2013 – 2014 seasons

**DOI:** 10.1038/s41598-018-34624-z

**Published:** 2018-11-15

**Authors:** Steffen De Vreese, Mike van der Schaar, Jürgen Weissenberger, Florence Erbs, Monika Kosecka, Marta Solé, Michel André

**Affiliations:** 1 Laboratory of Applied Bioacoustics, Technical University of Catalonia (UPC), BarcelonaTech, Rambla Exposición s/n, 08800 Vilanova i la Geltrú, Spain; 2 Statoil Norway, Notodden, Norway

## Abstract

While the Greenland and Barents Seas are known habitats for several cetacean and pinniped species there is a lack of long-term monitoring data in this rapidly changing environment. Moreover, little is known of the ambient soundscapes, and increasing off-shore anthropogenic activities can influence the ecosystem and marine life. Baseline acoustic data is needed to better assess current and future soundscape and ecosystem conditions. The analysis of a year of continuous data from three passive acoustic monitoring devices revealed species-dependent seasonal and spatial variation of a large variety of marine mammals in the Greenland and Barents Seas. Sampling rates were 39 and 78 kHz in the respective locations, and all systems were operational at a duty cycle of 2 min on, 30 min off. The research presents a description of cetacean and pinniped acoustic detections along with a variety of unknown low-frequency tonal sounds, and ambient sound level measurements that fall within the scope of the European Marine Strategy Framework (MSFD). The presented data shows the importance of monitoring Arctic underwater biodiversity for assessing the ecological changes under the scope of climate change.

## Introduction

Arctic waters are known to host a great biodiversity, although not much is known about the lifecycle of many species and how they depend on certain aspects of the ecosystem. The east coast of Greenland presents a continental shelf that transitions into very deep waters (>3 km) over a steep slope. As nutrient-poor cold water from the Arctic Ocean crosses Greenland’s continental shelf, nutrient-rich warmer waters are carried north by the Norwegian Atlantic Current into the Barents Sea and around Svalbard^1^. These conditions create upwellings that bring about nutrients and are associated with the seasonal abundance of fish and distribution of marine mammals^2^. Moreover, marine mammals show an intricate relation with the presence of sea ice, which, along the northeast coast of Greenland, consists of drift ice and icebergs carried from north to south by the East Greenland Current, and is usually very dense except for during the summer^3^. While some cetacean species, such as the beluga, prefer denser ice formations, others prefer more open waters with migratory species tending to migrate north in spring, when the ice starts to retreat. The area is also inhabited by pinnipeds, who rely on sea ice, e.g. as haul-out platforms when foraging^4^.

The Greenland and Barents Seas are populated by a variety of marine mammals that either migrate or are resident in the area, among them are whales of the Balaenopteridae family^5^. Although East Greenland waters are important for blue whales (*B. musculus*), there is hardly any research of this species in the area^5^. Both blue and fin whales (*B. physalus*) are migratory species, but very little is known about breeding or calving grounds and also the winter distribution is poorly understood. In warmer seasons, both species are known to migrate to northern waters, up to the ice edge. Blue whales have been seen as far north as Svalbard, while fin whales occur off the east coast of Greenland, mainly in summer and autumn^5^. The North Atlantic common minke whale (*B. acutorostrata acutorostrata*) is also known to occur as far north as Svalbard, at least in summer^6–8^, and also occurs along the southern half of Greenland^9^. All of these rorquals migrate south in winter, and north in summer^10^. As an exception, sei whales (*B. borealis*) do not tend to migrate as high north in summer and occur even further south in winter times^10^, but they have been spotted as far north as Iceland and in the Norwegian Sea^11^. Although humpback whales (*Megaptera novaeangliae*) have been spotted in East Greenland waters, there is very little known about their distribution and ecology. Between and around Svalbard and Iceland is one of the main feeding aggregations of North Atlantic humpback whales^5^. The bowhead whale (*Balaena mysticetus*), the only extant member of the *Balaena* genus, is an arctic resident species, and in the Greenland and Barents Seas, bowhead whales belong to the critically endangered Spitsbergen stock^12^. The only estimation for this stock is around one hundred individuals^13^ and there is a major lack of data regarding the seasonal distribution and migration pattern.

The species of toothed whale that can be present in the area covered by the recorders include sperm whale (*Physeter macrocephalus*), white-beaked dolphin (*Lagenorhynchus albirostris*), white-sided dolphin (*Lagenorhynchus acutus*), killer whale (*Orcinus orca*), northern bottlenose whale (*Hyperoodon ampullatus*), the *monodon* species narwhal (*Monodon monoceros*) and beluga (*Delphinapterus leucas*)^5,14^. Among pinnipeds that are known to occur in the Greenland and Barents Seas, there are the hooded seal (*Cystophora cristata*), harp seal (*Pagophilus groendlandicus*), bearded seal (*Erignathus barbatus*), ringed seal (*Pusa hispida*), harbour seal (*Phoca vitulina*), and the Atlantic walrus (*Odobenus rosmarus*). The first two undergo extensive seasonal migrations while the other species are residential to the area^5^.

Acoustic monitoring of the Arctic marine environment has been of increasing interest to the scientific community and management bodies^15–17^. The underwater soundscape, which is shaped by the traditionally measured physical acoustic signal and the dynamically changing acoustic environment, can be divided into natural (physical), biological and anthropogenic sources^18^. The last is mostly composed of shipping noise and sounds produced during oil and gas explorations. Natural sounds are those produced by e.g. the wind, the waves, and natural seismic activity while biological sounds come from marine life. All marine mammals are known to produce sounds as a primary means of communication, navigation, and foraging activities. Passive Acoustic Monitoring (PAM) provides a minimally invasive and very useful tool for monitoring underwater soundscapes and detecting the presence of marine mammals and other acoustically active marine life. This method can be used to measure their temporal and spatial distributions and moreover, allows for continuous monitoring of inaccessible environments such as ice-covered waters during the Arctic winter.

## Results

### Marine mammal detections

**Figure 1:**
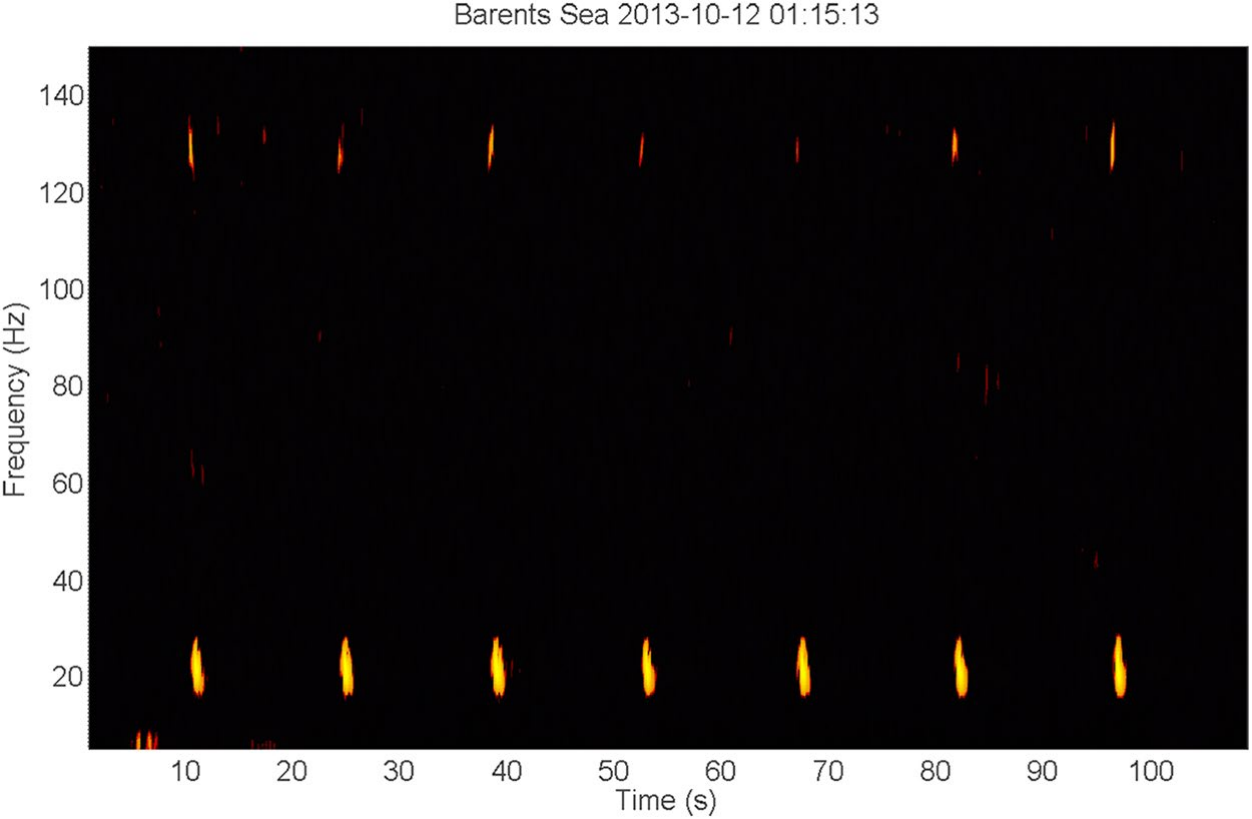
Spectrogram of a sequence of 20 Hz fin whale signals (time scale 2:00 min, linear frequency scale 0–150 Hz). The spectrogram shows the typical 20 Hz single downsweep signals, repeated every ±14 s. A higher frequency energy component around 127–131 Hz coincides with the temporal pattern of the 20 Hz signals, starting less than 0.5 s before the start of the latter and overlapping with it temporally.

In the Barents Sea, fin whale 20 Hz downsweep signals were present from the beginning of the deployment in October 2013^19^ (Fig. 1) to April 2014^20^ when only faint signals remained, and later also in August and September. At the Greenland I station, 20 Hz signals were detected in January^21^, February^22^, April^23^, May^24^, June^25^, July^26^, and August^27^. The months of September to December contained a variety of prominent noise in the low-frequency region that possibly inhibited the detection of fin whale signals. Similarly, the Greenland II station recorded a lot of anthropogenic noise from August to middle October 2013. There, possible fin whale signals were present in November^28^. Other species of the Balaenoptera genus might have been present as well, although not with absolute certainty. These species include minke whale (*B. acutorostrata*), sei whale (*B. borealis*) and blue whale (*B. musculus*).

Low-frequency tonal vocalizations attributable to bowhead or humpback whales were picked up by short tonal detectors operating in different frequency bands spanning from 100 Hz to 20 kHz. The calls sometimes contained energy from below 100 Hz, while the harmonics frequently ranged up to the recording limits, at least for the Greenland stations. There, bowhead whale songs were recorded from mid-October to mid-April (See Supplementary Information). Signals that match the typical temporal and frequency characteristics of bowhead whale calls were present over the entire recording period, although detections were rare during the summer months. The Barents data lacked these calls but similar signals were recorded from the beginning of November on^29^. The signals were faint until halfway December^30^, and the number of detections faded and disappeared in the month of March^31^.

Sperm whale clicks triggered the 5–20 kHz impulsive signal detector, and other impulse detectors operating below 5 and above 20 kHz. In the Barents Sea, there was sperm whale presence in October, December, January, March, and July. Manual inspection of the vocalizations detected in February^32^ could not confirm that these were produced by sperm whales. In Greenland I, sperm whale clicks were present every month over the entire recording period. Similar results were found for Greenland II, with the exception of no detections in August and September 2014. Rarely, but always temporally associated with sperm whale clicks, were various pulsed calls with often a tonal quality^33^.

Delphinid echolocation clicks triggered the high-frequency impulsive signal detectors (20–40 kHz and occasionally 5–20 kHz) in the Barents Sea only because the sampling rate of the Greenland recorders was too low to detect clicks with certainty. There were many strong occurrences in October, November, and sporadic events in December, March, and April. Whistles were confirmed in October, November, March, and April in the Barents Sea, while in Greenland I there was an especially high detection rate (8–20 kHz tonal detector) during winter, from December to February, followed by sporadic detections in March and April. No delphinids signals could be confirmed in the Greenland II data.

**Figure 2:**
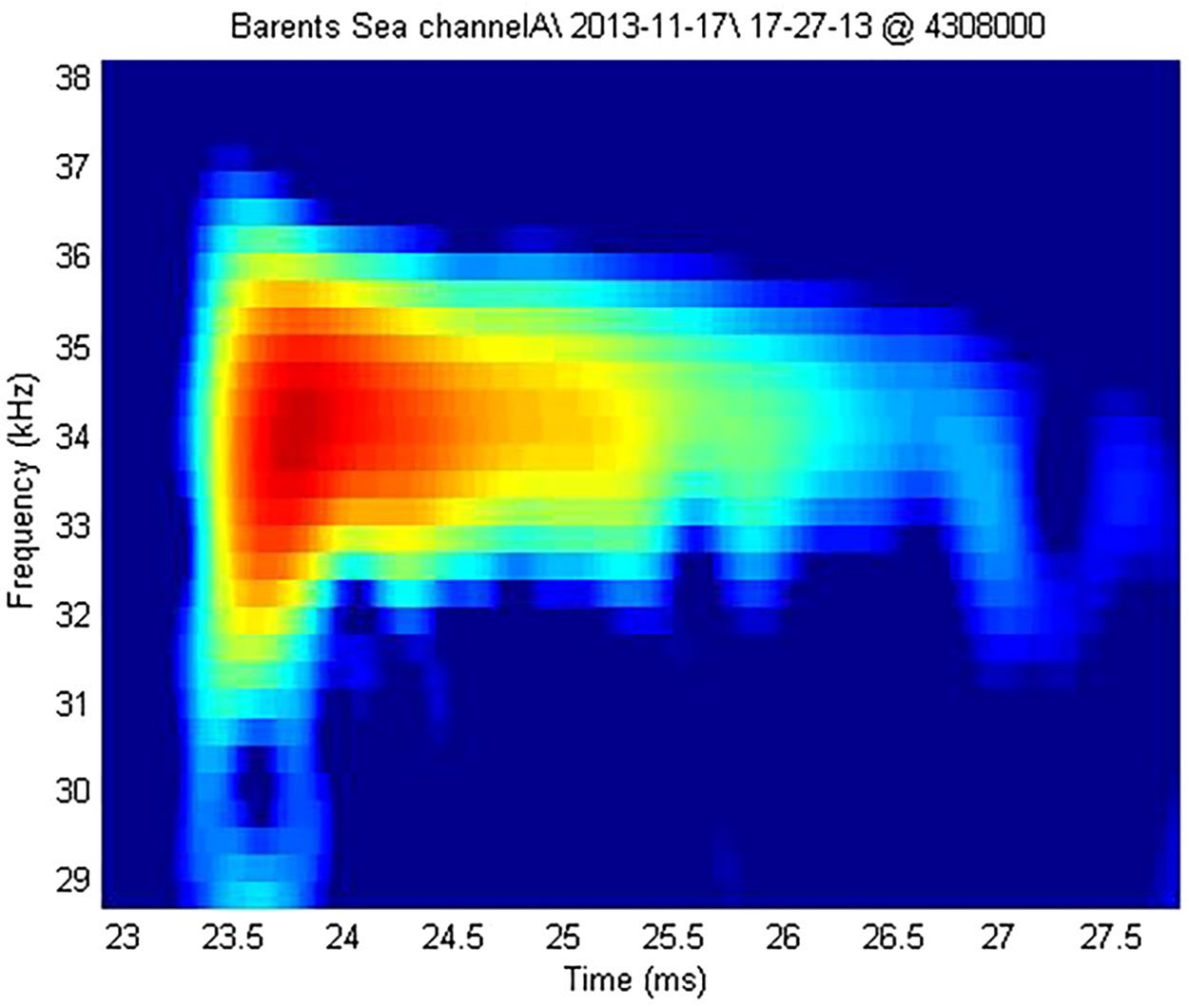
Spectrogram of a high-frequency echolocation pulse recorded in a click train on Nov 17, 2013. The image shows an indication of an upsweep, characteristic for beaked whale signals. The dominant frequency of all clicks in the segment lies between 31–39 kHz above which the signal could not be analyzed qualitatively (Nyquist frequency).

Beaked whales may have been present in the Barents Sea in November (Fig. 2). If the signals were produced by beaked whales, it is likely to be either a Northern bottlenose whale (*Hyperoodon ampullatus*) or Sowerby’s beaked whale (*Mesoplodon Bidens*) based on limited information of their acoustic signals and geographical distribution^34,35^. However, the limited sampling rate of the recording did not permit a definite conclusion.

Bearded seal trills and other tonal sounds were predominantly detected in the 1–8 kHz frequency range. Detectors operating in the 8–20 kHz, 150–900, and 250–1500 Hz bands triggered towards the beginning or the end of the signal respectively, as it entered the according frequency band (See Supplementary Information). In the Barents Sea, the first records of bearded seal trills started on April 2^36^, continued until April 10, and were detected every day from April 27 to July 1. In Greenland I, trills were recorded the first days of April, were highly abundant in May and June, and suddenly dropped in the beginning of July. The same was found on the Greenland II recordings, except that the first recordings occurred in the second week of February. In all three stations, the presence of trills suddenly dropped at the beginning of July. Other pinniped vocalizations were found in the Barents sea on various occasions^37–39^. No attempt was made to distinguish between species.

Moreover, a variety of delphinid pulsed calls and tonal calls was recorded at all three stations, although most prominently in the Barents recordings. These are most likely to be produced by narwhal, beluga, or killer whale, but no attempt to distinguish between these species is presented in this paper. Here we present some examples of unclassified sounds^40–47^.

An overview of the different taxon’s acoustic presence can be seen in Table 1.

**Table 1:** Overview of the different species’ acoustic presence found at the three stations: Barents Sea (B), Greenland I (G1), and Greenland 2 (G2).

Taxon	Site	2013				2014									Vocalizations
		S	O	N	D	J	F	M	A	M	J	J	A	S	
*B. physalus*	B	/	**+**	**+**	**+**	**+**	**+**	**+**	**+**				**+**	**+**	20 Hz signals
	G1					**+**	**+**		**+**	**+**	**+**	**+**	**+**		
	G2			**+**											
*m. novaeangliae*	B	/													Calls and songs
	G1														
	G2														
*B. mysticetus*	B	/													Calls and songs
	G1		**+**	**+**	**+**	**+**	**+**	**+**	**+**						
	G2				**+**	**+**	**+**	**+**	**+**						
*p. macrocephalus*	B	/	**+**		**+**	**+**	**+**	**+**				**+**			Clicks
	G1		**+**	**+**	**+**	**+**	**+**	**+**	**+**	**+**	**+**	**+**	**+**	**+**	
	G2	**+**	**+**	**+**	**+**	**+**	**+**	**+**	**+**	**+**	**+**	**+**			
*Lagenorhynchus*	B	/	**+**	**+**	**+**	**+**	**+**	**+**	**+**						Clicks and whistles
	G1				**+**	**+**	**+**	**+**	**+**						
	G2														
*Monodontidae*/*O. orca*	B	/						**+**	**+**	**+**		**+**	**+**		Clicks, pulsed calls and whistles
	G1														
	G2														
Ziphiidae	B	/		**+**											Frequency modulated clicks
	G1														
	G2														
*E. barbatus*	B	/							**+**	**+**	**+**	**+**			Trills
	G1								**+**	**+**	**+**	**+**			
	G2								**+**	**+**	**+**	**+**			
Other pinnipeds	B	/							**+**	**+**	**+**	**+**			Tonal vocalizations
	G1														
	G2														

Only months with confirmed positive detections are highlighted with a plus mark. Unmarked cells signify that the taxon could not be assigned with certainty. The ‘/’ mark for the Barents site means there was no recording in the month of September. The ‘X’ marked cells indicate there was no distinction between the two species of baleen whales.

### Unknown low-frequency tonal signals

**Figure 3:**
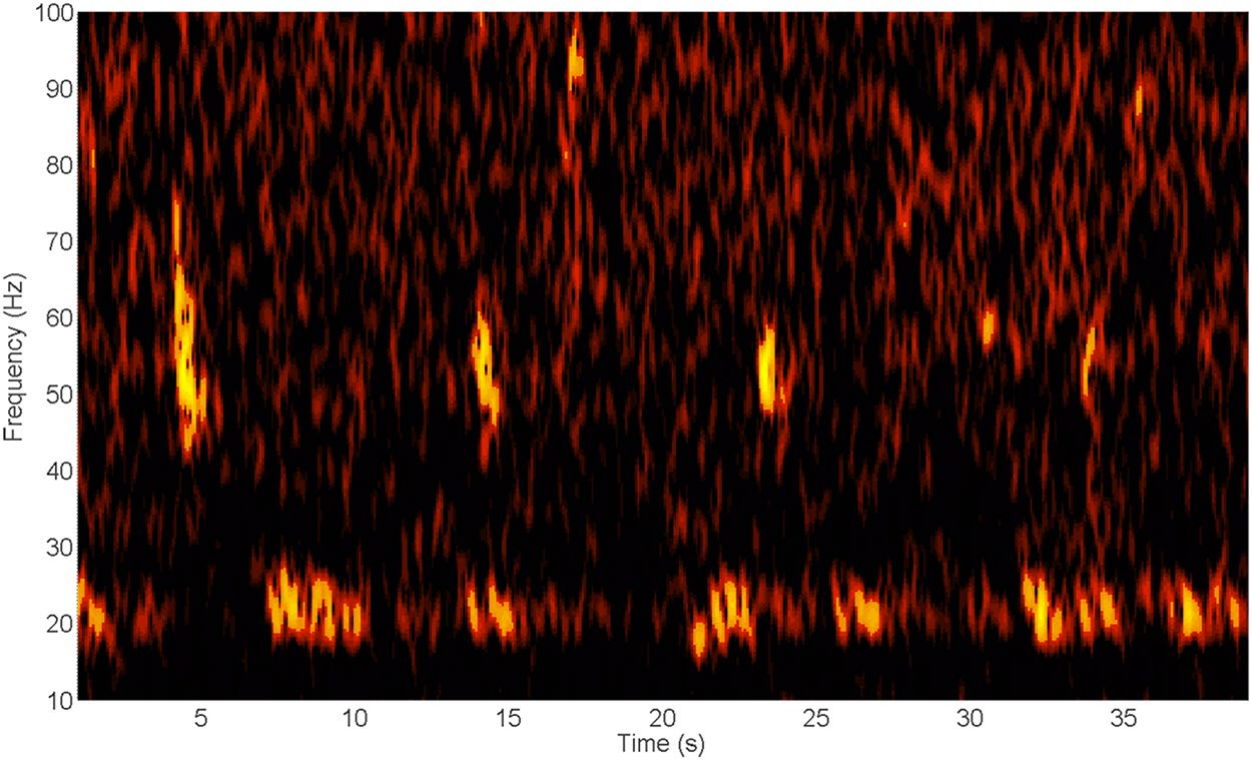
Spectrogram demonstrating the presence of various low-frequency tonal sounds (time scale 120 s, frequency scale 0–120 Hz). There are typical 20 Hz fin whale signals. Higher in frequency are FM downsweep signals from 80 to 40 Hz with most dominant energy between 50–60 Hz. These signals last about 1–1.5 s, starting with a relatively broadband part of ±1 s, with the low-frequency part continuing in a downsweep of ±0.5 s ending at about 50 Hz. They occur at an interval of about 9 s. Concurrently, there are constant frequency tonal signals with energy just above 3 Hz and a probable harmonic just above 6 Hz. The signal duration varies from about 5 to 10 s. The interval between latter sounds is highly variable, with a period of silence of at least 3 s between signals^101^.

**Figure 4:**
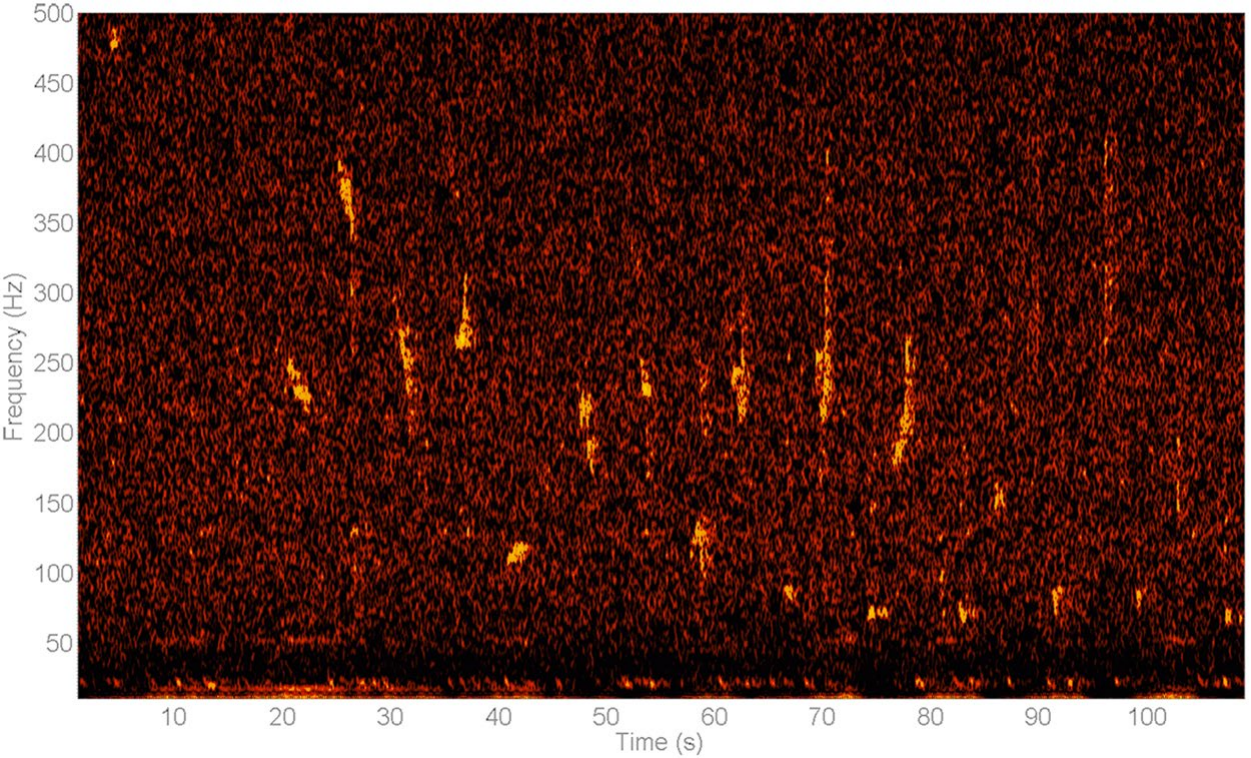
Spectrogram of a variety of tonal signals occurring between 50 and 400 Hz (Timescale 90 s, frequency scale 10–560 Hz). A similar pattern of alternating high and low calls was noted on other occasions (See Fig. 7). Note the concurrent presence of 20 Hz fin whale signals^102^.

**Figure 5:**
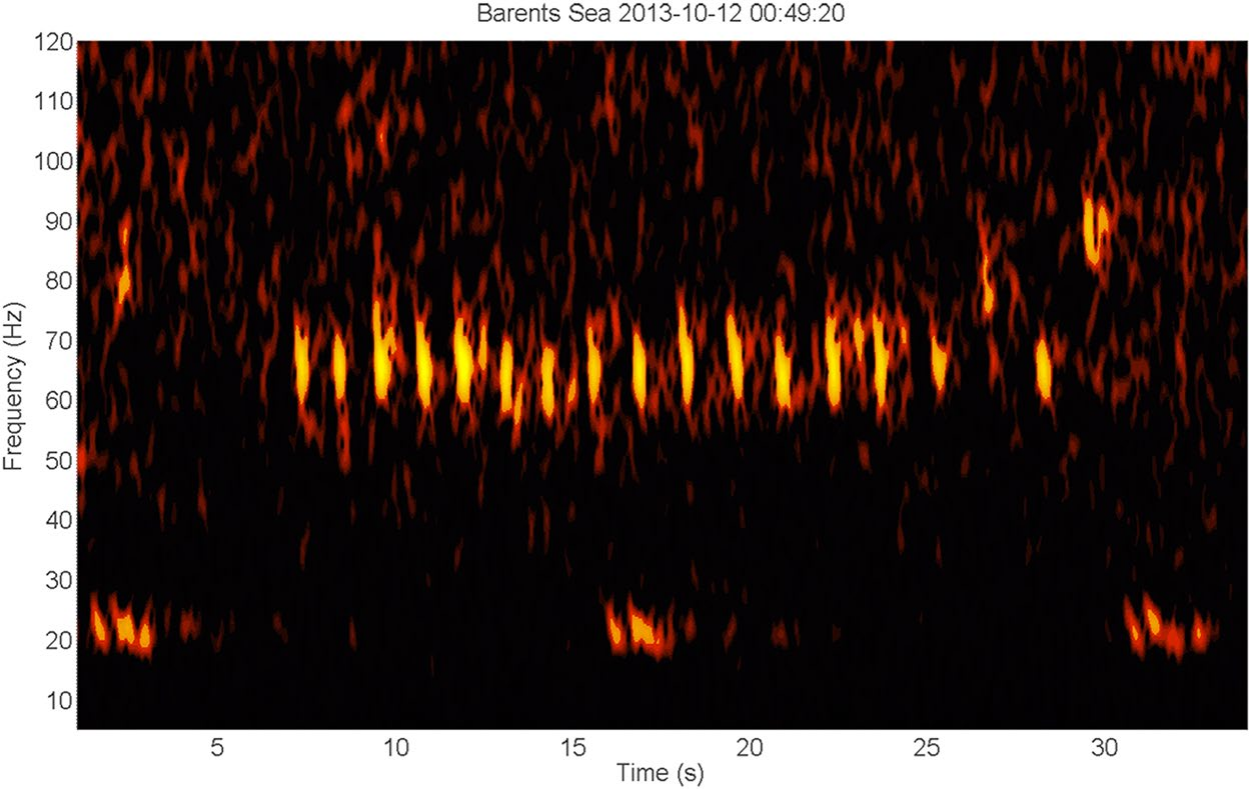
Spectrogram of a series of tonal downsweep signals of unknown origin^103^. (Timescale 28 s, frequency scale 50–105 Hz). This series constitutes 16 low-frequency slightly FM downsweep signals between roughly 60–70 Hz, and with a duration of 0.6–0.7 s. The first 15 signals occur with an interval of 1.0–1.2 s, and are followed by a faint higher frequency signal with energy around 76 Hz, followed by another ‘normal’ signal, followed by a signal in frequency band 82–94 Hz, followed by a silence of 5.6 s, and ending with a faint signal at 87 Hz. There were concurrent 20 Hz fin whale signals with additional energy at 130 Hz, and also very low-frequency tonal signals around 3 and 6 Hz.

**Figure 6:**
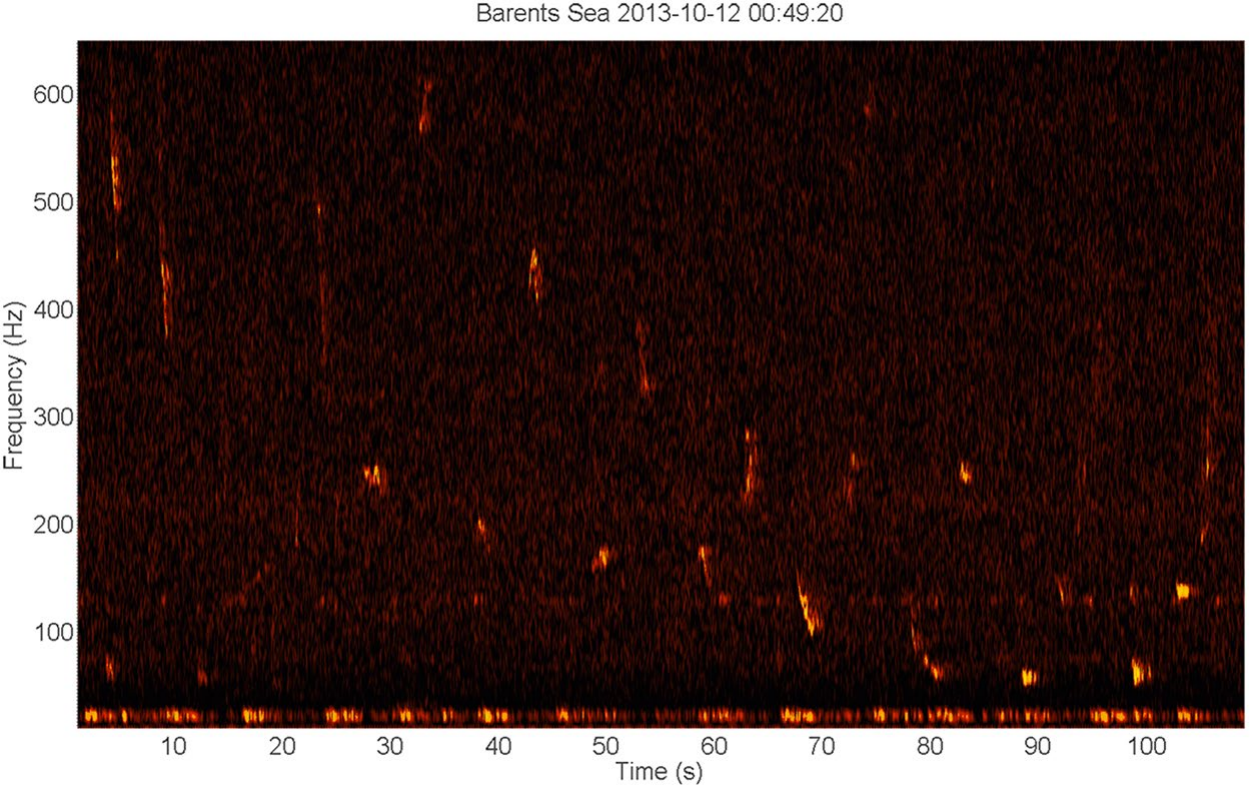
Spectrogram of unknown tonal signals in a sequence of about 2 min (Timescale 110 s, frequency scale 10–650 Hz). The signals are short, frequency modulated down- and upsweep signals occurring in a sequence that starts at about 400–600 Hz and ends around 50 Hz. There is a clear alternating pattern between low and higher frequency units^5^ (Similar signals were detected on several days at the end of October (e.g.^104–107^).

**Figure 7:**
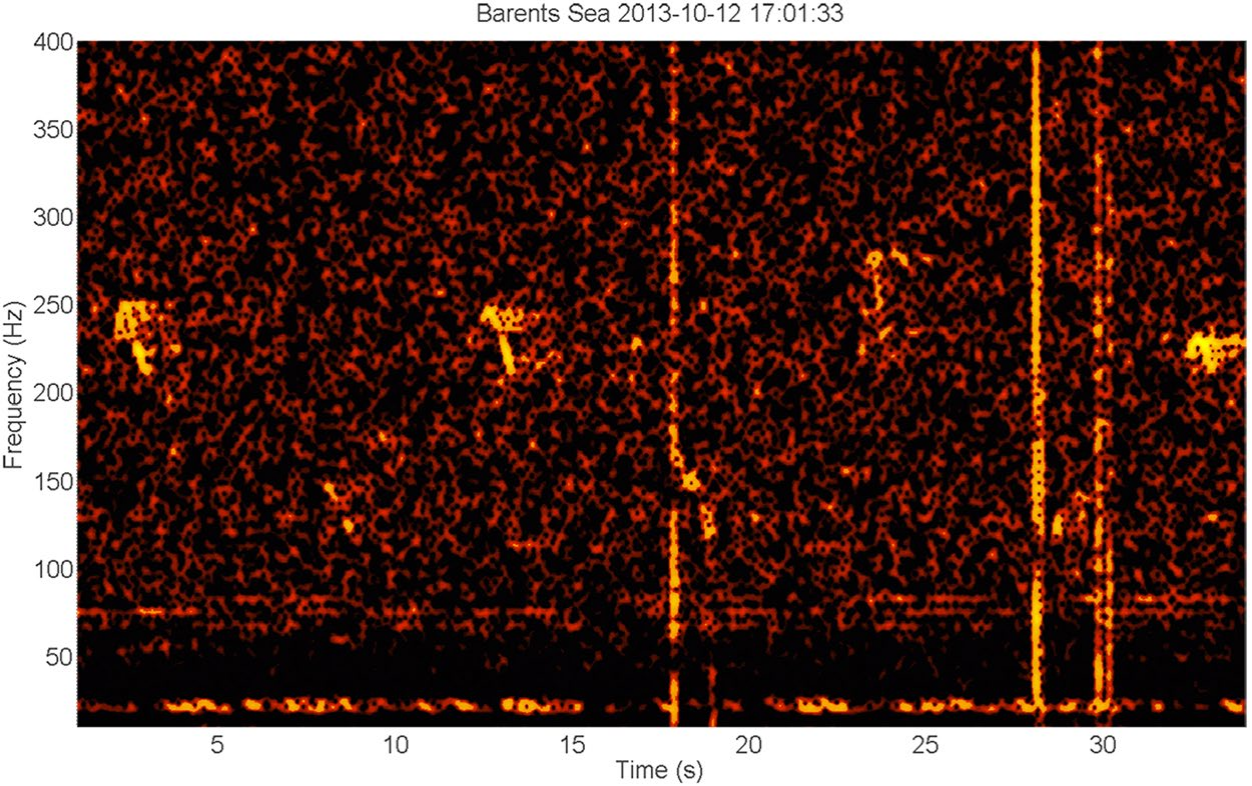
Spectrogram (time scale 35 s, frequency scale 10–400 Hz) of tonal arch sounds with dominant frequency around 220–230 Hz^108^. These signals are similar to LF arch sounds attributed to blue whales^109^.

A variety of unknown tonal signals were found at all three stations although most prominently in the Barents recordings. Although many of these vocalizations could probably be attributed to pinnipeds or cetaceans, more research is needed on the vocal repertoire of many arctic species. Figures 3–7 depict several tonal signals from unknown sources.

On multiple occasions, and particularly in October and November 2013, the Barents Sea station recorded unknown tonal sounds of lower SNR, such as these whistle-like sounds around 4–5 kHz followed by LF tonal sounds around 400–500 Hz, and concurrent fin whale signals^48^. The Greenland I station also recorded miscellaneous baleen whale sounds such as moan- and ‘gunshot’-like sounds^49^.

### Anthropogenic noise

**Figure 8:**
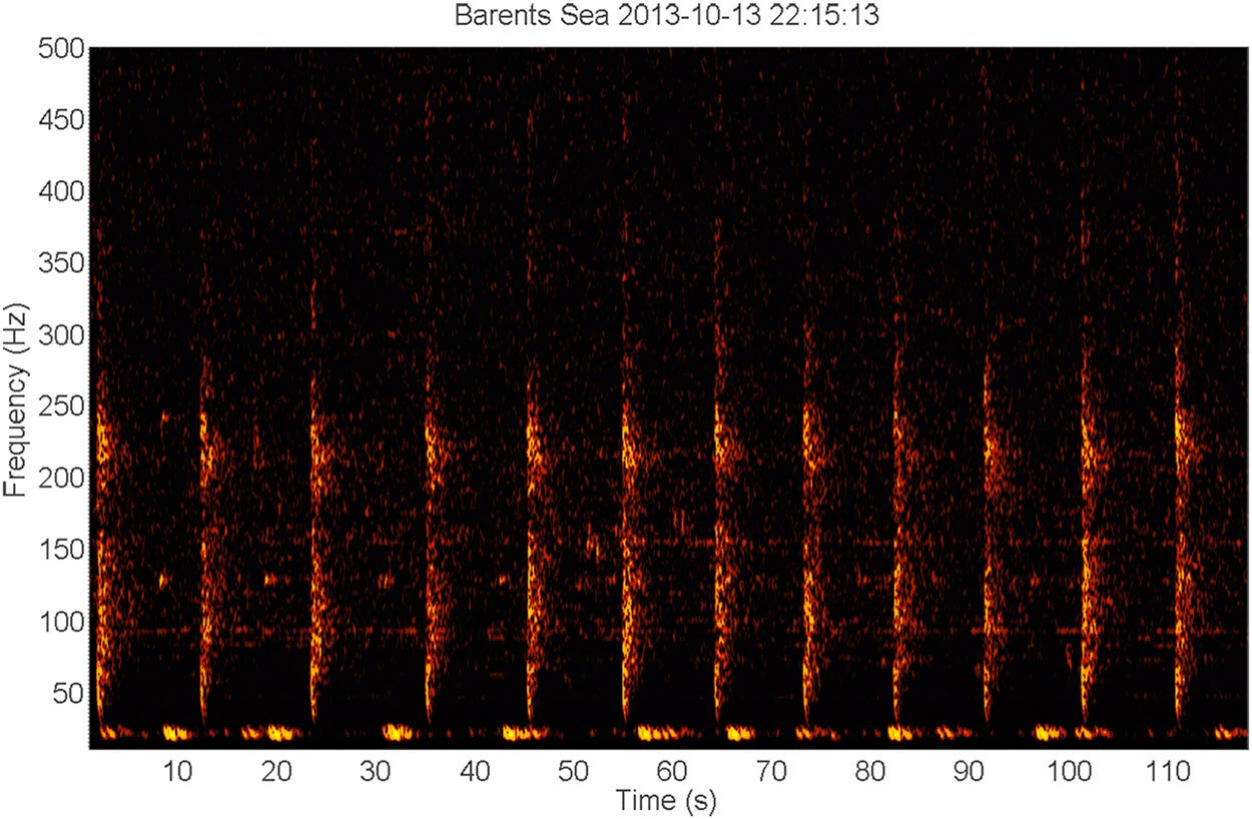
Spectrogram of 20 Hz fin whale signals coinciding with pulses from a seismic survey. (Hann window, spectral resolution 4096, timescale 2:00 min, logarithmic frequency scale 10–700 Hz). The spectrogram shows the 20 Hz double downsweep signals, repeated every ±14 s. Although less clear, there seems to be higher frequency energy around 127–131 Hz coinciding with the temporal pattern of the 20 Hz signals, occurring less than 0.5 s before the start of the latter, with a harmonic around 260 Hz.

A variety of anthropogenic noises was found in the recordings. Airgun triggers from several seismic surveys were detected by all impulse detectors operating below 5 kHz^50^. Especially in the months of November, and January to May, the Greenland II site presented many sounds from seismic surveys, while both Greenland stations showed seismic activity in late summer. At the Barents stations seismic surveys were present in September and October 2013 (Fig. 8), and March, April and July 2014. The 5–20 kHz detector picked up anthropogenic noise produced by the presence of ships with a peak in July and August. Other mechanical sounds could possibly be attributed to anthropogenic activity in the area^51^, or to self-noise^52^.

### Other notable sounds

Moving or breaking ice triggered the mid- and low-frequency impulse detectors (1–20 kHz) while whistling ice was detected by several short tonal detectors active in the 1–20 kHz band. All recordings contained sounds of breaking^53^ and whistling ice^54^, with the highest presence in the northern Greenland Sea recorder. This corresponds to satellite data that showed that the northern recorder was always encircled by drift ice, although less so in summer, while the southern recorder was situated in relatively open waters during August and September. The situation in the Barents Sea was similar to the latter, with open waters in summer and relatively open to close drift ice in winter^55^.

In Greenland I, an unknown sonar signal around 6–7 kHz that repeated every 1–1.5 s, was present for several days in September^56^, October^57^, November^58^, January^59^, February^60^, March^61^, May^62^ and June^63^. Although there is some resemblance to sperm whale clicks, the highly regular nature of this signal and its almost constant presence is not indicative of sperm whale clicks. The same signal was also found in the Barents recording, at least during the month of May.

The system often produced low-frequency impulsive self-noise^64^ another example of noise of unknown origin can be heard here^65^.

### Sound levels

The sound pressure levels in the third-octave bands centred at 63 Hz and 125 Hz were measured in the scope of the Marine Strategy Framework Directive (2008/56/EC) (D11.2). Mean values, computed over almost the entire recording period and leaving out 1% of the highest snapshot sound pressure levels, were 92 and 96 dB re 1 μPa^2^ respectively for the Barents Sea, 100 and 94 dB re 1 μPa^2^ for the Greenland I site, and 99 and 95 dB re 1 μPa^2^ for the Greenland II site (see^66^ for a detailed analysis of a seismic survey analysis).

## Discussion

The use of passive acoustic monitoring proved to be very useful in the detection of marine mammal vocalizations in the Greenland and Barents Sea. The diversity in the recorded calls was considerable, which indicated the presence of many species but also made it difficult to identify with certainty which species were present during which period. The data clearly indicated both a spatial difference between recording sites, as well as a seasonal difference within the recording period. As such, cetaceans such as sperm whale and bowhead whale might be present all year round, with peaks in vocal behaviour during winter, while other species such as humpback whale might exhibit a seasonal presence.

In this study, we only rely on true acoustic cues as an indicator of physical presence. The absence of detections does not necessarily indicate the absence of a species as it might just be a temporal trend in the vocal behaviour, which in itself could reflect a form of social communication. That is to say, a given individual may remain silent for some time and thus remain undetected from the observatory but is therefore not absent. For example, the bearded seal trills are only produced by males in a mating context^67^. Moreover, a higher signal detection or vocalization rate does not necessarily imply a higher absolute abundance of individual animals. For example, sperm and bowhead whales do not necessarily have a higher relative abundance during winter, as the higher vocalization rate could be related to a specific behaviour^68^.

**Figure 9:**
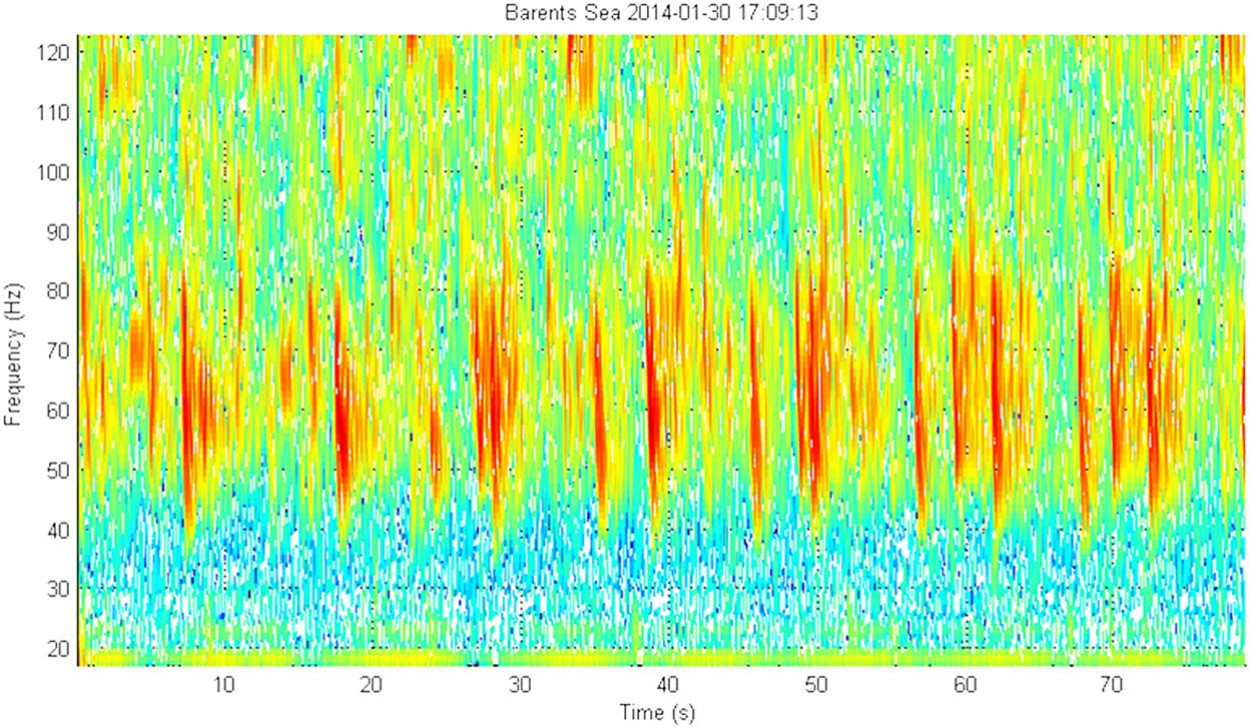
Spectrogram of multiple repetitive downsweeps recorded in the Barents Sea on January 30, 2014^110,111^. (Timescale 80 s, linear frequency scale 10–120 Hz). The signals start at about 80 Hz and sweep down to about 45 Hz. There is a clear distinction between three downsweep signals that each have a specific start and end frequency. Each of the three downsweeps repeats every 9–11 seconds. These signals were recorded in concurrence with very faint 20 Hz fin whale signals that repeated every ±15 seconds. In the same run, there were concurrent higher frequency probable humpback whale signals and faint sperm whale clicks.

The fin whale’s most common vocalizations are trains of the ‘classic’ 20 Hz signals, which are signals that sweep down from about 30 to 20 Hz over a duration of about 1s^69^. These are sometimes associated with backbeat pulses or higher frequency components^70,71^. They can also produce other tonal calls such as higher frequency downsweeps (<100 Hz)^72,73^ (Fig. 9). A thorough manual analysis was needed to reveal the temporal patterns in the presence of their typical vocalizations. The Barents recordings contained fin whale signals in winter, spring and late summer, while the Greenland I recordings indicated a presence of fin whale from January on. However, it cannot be excluded that the whales were not present before this time as there was significant low-frequency noise hindering possible detections. Although not fully certain, our results point towards a year-round presence of fin whale in these arctic waters. This concurs with Clark’s^74^ acoustic data that pointed out that fin whales may be found in their entire range in all seasons. Furthermore, the manual analysis may constitute some false negative results as it was sometimes difficult to distinguish fin whale calls from distant seismic activity. To help to differentiate these signals, the specific frequency and temporal characteristics, such as the start and end frequencies, the signal duration, as well as the length and consistency of the intervals between signals were studied in detail. Similarly, sei whale have been known to produce downsweep signals that are similar in frequency and duration although this species has not been reported as far north as the Svalbard archipelago^75,76^. Moreover, minke and blue whale have been attributed similar vocalizations^77,78^, both of which do occur in Arctic waters, but here, no vocalizations could be attributed to any of these species with certainty.

Bowhead and humpbacks whales are the two only mysticete species known to display complex and elaborate songs. There is an important overlap in the frequency range of bowhead and humpback whale vocalizations, both covering a continuum between low and high frequencies ranging from 20–30 Hz up to several kilohertz^79,80^. Thus, the acoustic distinction between these two species is not always clear. Particularly, this overlap in frequency parameters did not allow frequency-based automatic detectors to detect these species apart. However, manual inspection can help assess whether the vocalizations belong to one or the other species based on the complexity and duration of the songs. In comparison to humpback whale songs, the song sequences of bowhead whales appear slightly less complex, including a lower number of different units per song, which are also shorter in duration^81^. In addition, the simultaneous production of two harmonically unrelated sounds (biphonation) has been confirmed in bowhead whales^82^ but has not yet been described for humpback whales. The presence of this remarkable feature in the recordings from Greenland is an additional and strong indication that the recorded songs are from bowhead whales. The detection of considerable singing activity from this species from the Northeast Greenland coast is in accordance with a recent acoustic study from Stafford *et al*.^83^. This study reported a similar seasonal pattern of song detection in the Fram Strait, close to the Greenland recording sites of this study. The results presented here concur with data from recent sighting surveys^13,84^. The analysis of a subset of the recordings from the Barents Sea did not reveal vocalizations that could be attributed to either bowhead or humpback whales with certainty. An extensive inspection of the data could bring additional information on the presence of this species in this area.

Sperm whale produce short and broadband pulsed sounds at various repetition rates. The usual clicks occur with regular, or regularly changing, inter-click interval of 0.5 to 2.0 seconds^85–89^ while other sounds can constitute clicks occurring in a very rapid succession and can even reach tonal qualities^90,91^. Most energy lies in the sonic region but the signals can have energy higher than 32 kHz^92^. The Greenland I station was situated about 120 km from the continental slope, which is where sperm whale activity was expected, and proved useful for detecting sperm whale clicks, all year round. The clicks were detected well by the 5–20 kHz impulse detector when the signal to noise ratio was not too low, but the output was occasionally mixed with impulsive shipping noise. A combination of detectors was used to eliminate shipping and to obtain regions of interest for manual inspection. Occasionally, there were pulsed and tonal calls that were temporally associated with known sperm whale sounds such as the ‘trumpet’ sound or yelps, squarks, chirps, pips, or squeals^90,91,93^. The impulse sounds recorded in the Barents Sea in February were not clearly received, and resembled signals from distant geophysical prospecting, but the rhythmic pattern indicated that these were more likely produced by sperm whales.

Smaller odontocetes were recognized by the detection of echolocation pulses, whistles, and/or pulsed calls. No distinction was made between the pulsed calls of narwhal, beluga and killer whale calls in any station. Although beluga can occur in all Arctic waters, it has been only rarely seen in the Greenland Sea^5^. Several whistles recorded in the Barents Sea could be attributable to white-beaked dolphin based on their spectrographic similarity to whistles described by Rasmussen *et al*.^94^. Moreover, the whistles recorded in March and April at Greenland I might also have been produced by white-beaked dolphins, and it cannot be excluded that other delphinid species such as Atlantic white-sided dolphins were recorded as well. Preliminary results point out that there are several distinct types of whistles reoccurring in the data, and more research is needed to investigate this further. Also, the recording of possible beaked whale signals in the Barents Sea indicate that more acoustic studies in that region could be useful, although a high sampling frequency would be required.

Male bearded seals have been known to produce distinctive trills as a breeding display^95^. Detections peaked in May and June, with much lower detection rates in April and July as corresponding to the breeding season and to previous acoustic studies in the area^96^. Moreover, it was remarkable to find that the detection of trills suddenly started and stopped as if from one day to the next, with the exception of a few faint signals. No signals could be attributed to other pinniped species with certainty, and more information is needed on their underwater vocal repertoire. Although walruses have been known to occur in the vicinity of the Greenland recorders^5^, no vocalizations could be attributed to them with certainty.

**Figure 10:**
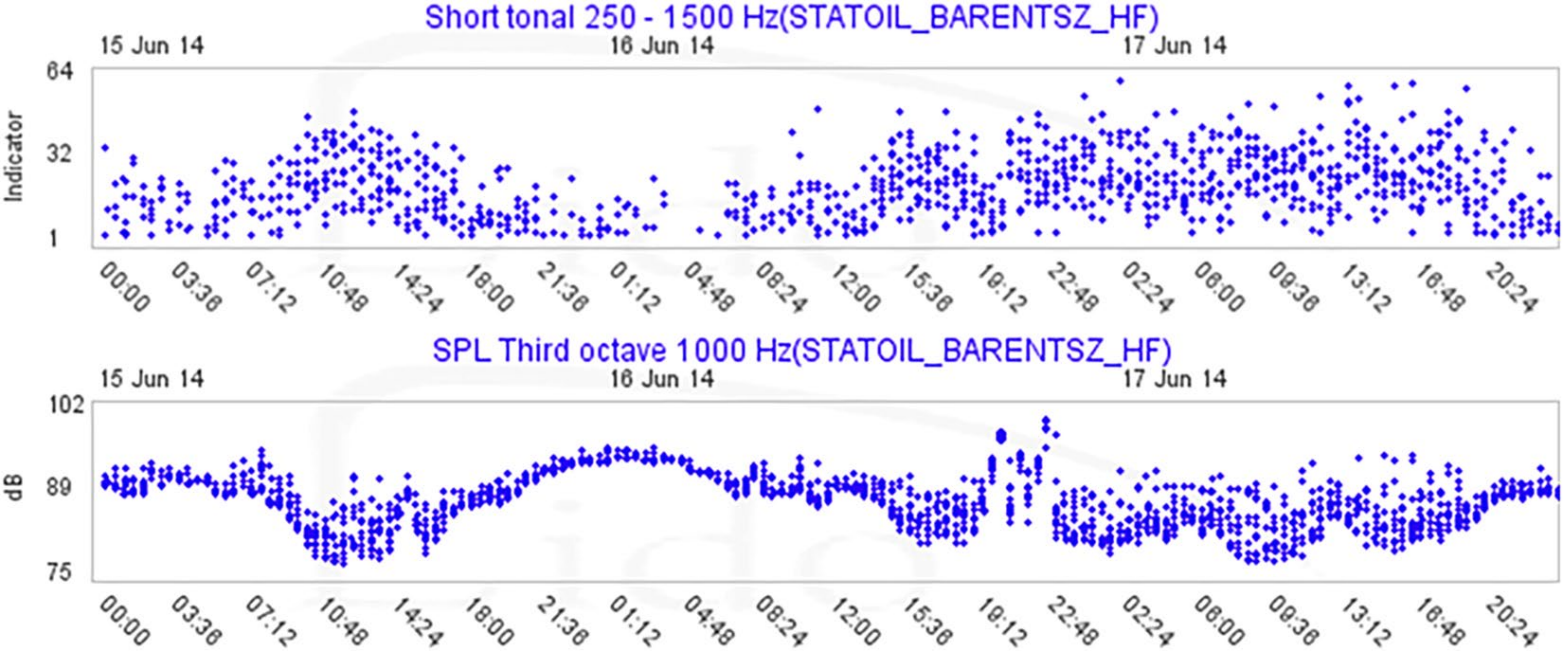
Output of the 250–1500 short tonal detector (above) and concurrent SPL of the third-octave band centred at 1 kHz (below). Each dot represents an analyzed segment (For detailed description, see^97–99^). The image shows a drop in tonal detections when there is a rise in the amount of background noise. This visual correlation corresponds to the signals being masked by noise coming from a passing ship on the night of June 15th. Apart from a reduction in detection range, the increase in anthropogenic activities itself may have caused a change in behaviour, or the animals may have moved with the change in ice although the latter is less probable as the same types of tonal signals (bearded seal trills) were detected before and after the increase in noise.

Over the entire recording period, there were visual indications of the correlation between the amount of noise and the number of automatic marine mammal detections in overlapping frequency bands (e.g. Fig. 10). Accordingly, the automatic detection results varied depending on the signal to noise (anthropogenic, natural and self-noise) ratio and the nature of the signals of interest.

The presence of airgun pulses during several months of the recording period in the Greenland stations did not interfere with the general detection of any marine mammal calls because the repetition rate of the pulses was such that even low-frequency calls could be detected between pulses. However, these noises did complicate the detection of fin whale 20 Hz signals in that there could be masking when the signals coincided temporally.

All three stations reported extensive anthropogenic activity related to seismic surveys. However, since the repetition rate of the airguns was fairly low (~10 s), the detectors themselves did not give high outputs since impulse detector output takes into account the weighted number of detected impulses within a segment. Moreover, shipping noise was present on all three stations, with the most prominent activity in summer. This is most likely related to the level of sea ice coverage.

As the Arctic ambient soundscapes are very little known, it is essential to gather baseline acoustic data employing consistent and uniform methodologies. A fundamental focus should include basic sound level measurements and species call detection and identification. The results of this research confirm the great biodiversity in Arctic waters, as they indicate the year-round presence of a large variety of marine mammals in the Greenland and Barents Seas, with species-dependent seasonal and spatial variation. As offshore anthropogenic activity increases, it is essential to monitor and assess the impact this might have on the marine environment and the animals that inhabit it. This study illustrates that passive acoustic monitoring (PAM) is a useful and non-invasive tool for the long-term monitoring of acoustic conditions and related ecosystem biodiversity. This information is vital for understanding and managing the ecological transformations that occur in the Arctic under the scope of climate change.

## Methods

### Acoustic recorders

**Figure 11:**
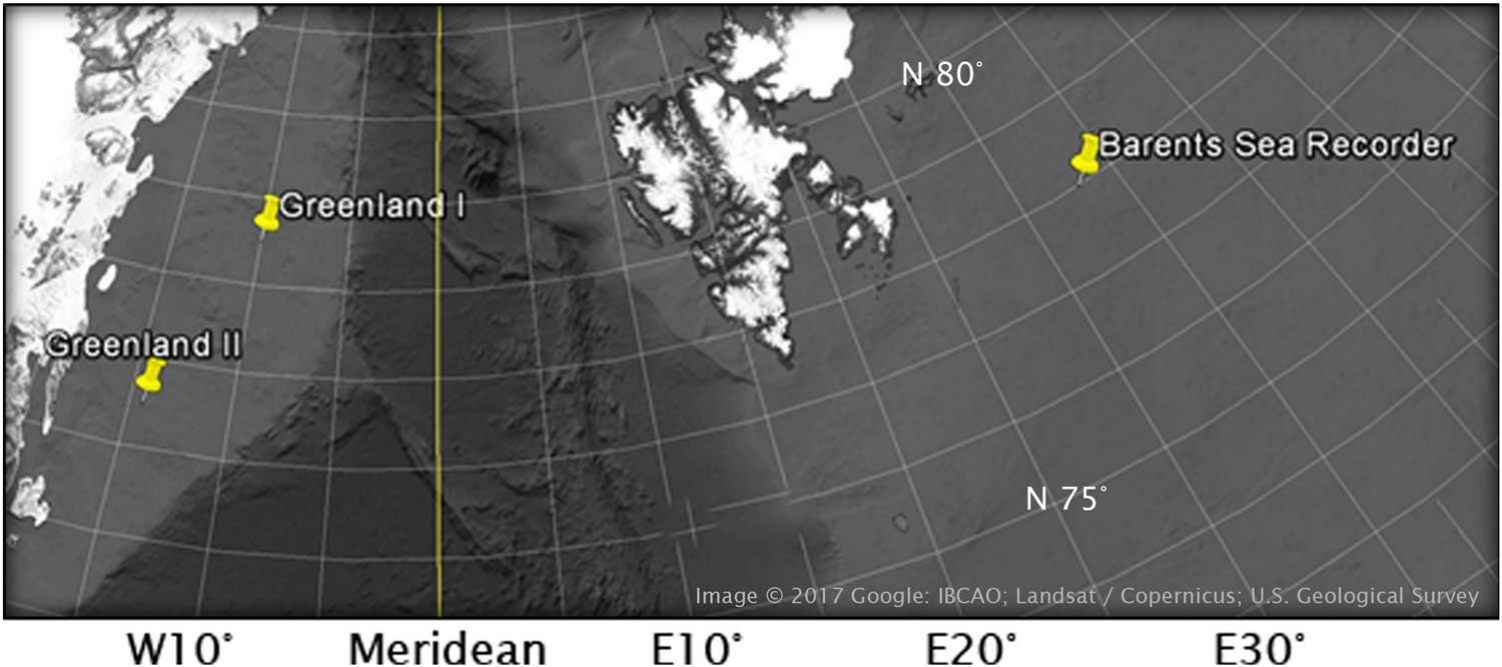
Acoustic recorder locations in the Greenland and Barents Sea. Part of Greenland’s northeast coast is visible on the left side. The land mass in the top centre of the image is the Norwegian archipelago Svalbard.

As part of the ODEN Arctic Technology Research Cruise 2013, four recorders were deployed in the fall of 2013, two at the continental shelf northeast of Greenland (78°30’N, 10°00’W and 76°30’N, 14°20’W) and two in the Barents Sea west of Svalbard, and recovered again in the fall of 2014, except for one recorder in the latter location (Fig. 11). Water depth was about 200 m at all locations and recorders were bottom-moored, floating approximately 50 m above the seafloor. The Greenland site system was fitted with AQUATech Low Power Scientific Measurement hydrophones with sensitivity −160 dB re 1 V/µPa. Data was sampled at 39062 Hz with 24-bit resolution, quantized between +−2.5 V, and using a recorder gain setting of −0.732 and −0.576 dB in Greenland I and II respectively. The Barents *site* was equipped with an HTI-96 hydrophone (High Tech Inc., Gulfport, MS, USA) with sensitivity −170 dB. Acoustic data was recorded at a sampling rate of 78125 Hz with 24-bit resolution, quantized between +−2.5 V, and with the gain set at −0.588 dB. All systems were operational at a duty cycle of 2 min on, 30 min off. See Table 2 for more details.

**Table 2:** Technical specifications of the recording equipment.

Name	Greenland Sea I	Greenland Sea II	Barents Sea
Hydrophone	AQUATech Low Power Scientific Measurement	AQUATech Low Power Scientific Measurement	HTI-96 (High Tech Inc., Gulfport, MS, USA)
Sea	Greenland Sea	Greenland Sea	Barents Sea
Coordinates	78°30′N, 10° 0′W	76°30′N, 14°20′W	77° 0′ N, 32°59′ E
Start of recording	23/08/2013	22/08/2013	03/10/2013
End of recording	17/09/2014	17/09/2014	17/10/2014
Water depth	−223 m	−201 m	−189 m
Sampling rate	39062 Hz (24 bits, +/−2.5 V)	39062 Hz (24 bits, +/−2.5 V)	78125 Hz (24 bits, +/−2.5 V)
Sensitivity	−160 dB re 1 V/µPa	−160 dB re 1 V/µPa	−170 dB re 1 V/µPa
Recorder gain	−0.732 dB	−0.576 dB	−0.588 dB
Duty cycle	2 min on – 30 min off	2 min on – 30 min off	2 min on – 30 min off
Detectors	Short tonal (10–45 Hz; 150–900 Hz; 500–2500 Hz; 1000–8000 Hz; 8000–20.000 Hz) Impulse (15–25 Hz; 250–650 Hz; 500–5000 Hz; 2–5 kHz; 5–20 kHz)	Short tonal (10–45 Hz; 150–900 Hz; 500–2500 Hz; 1000–8000 Hz; 8000–20.000 Hz) Impulse (15–25 Hz; 250–650 Hz; 500–5000 Hz; 2–5 kHz; 5–20 kHz)	Short tonal (10–45 Hz; 100–300 Hz; 250–1500 Hz; 1000–8000 Hz; 8000–20.000 Hz) Impulse (15–25 Hz; 250–650 Hz; 500–5000 Hz; 5–20 kHz; 20–46 kHz)

### Analysis methods

Sound levels were measured and marine mammal vocalizations were automatically detected using the SONS-DCL software package^97–99^, which has been developed in the scope of the LIDO programme^100^. It includes modules for sound level measurements, acoustic event detection and classification, localization, spectrogram creation, acoustic data compression and other functions. The output of the automated detectors, described below, was analyzed, although a complete manual analysis was performed when results were suspected to be unreliable.

#### Detection procedure

The raw acoustic data was processed in consecutive, non-overlapping segments of about 13 seconds. In each station, five short tonal and five impulsive signal detectors were used, with each detector operating in a specific frequency band, altogether covering the entire recording bandwidth (>10 Hz). The lower frequency short tonal detectors aimed at detecting tonal signals from baleen whales such as bowhead whales, humpback whales, and species from the Balaenopteridae family. The low and mid-frequency detectors also aimed at detecting pinniped vocalizations. Higher frequency short tonal detectors were used for detecting tonal sounds from the Delphinoidea superfamily and were also responsive to pulses occurring in a very rapid succession. High-frequency delphinid echolocation clicks could be detected by the ultrasonic impulse detector while the lower frequency impulse detectors (such as in the 5–20 kHz band) were useful for detecting sperm whale clicks and shipping noise. Several low-frequency impulse detectors served to pick up a specific noise coming from the system. The output of these detectors was used as an elimination filter to look for interesting segments.

The output of the automatic detection process was used as an indication for manual analysis through the LIDO interface. Spectrograms were created automatically covering the full bandwidth of the recorders (up to 20 or 40 kHz, respectively) and additional lower frequency spectrograms were created (0–5 kHz; 0–900 Hz; 0–150 Hz) to provide a better view of lower frequency signals. The user interface also provided a playback feature with a compressed audio stream and moving spectrogram. Regions of interest for manual inspection were found using information from the detectors combined with sound level measurements. For example, moments with a high number of detections would be interesting for manual inspection while moments with high noise levels were likely not of interest. After identifying regions of interest, segments were selected at random checked for marine mammal presence. Moreover, uncompressed data was analyzed by means of a standard audio processing software for closer inspection in time and frequency bands of interest. The recorded data was examined both visually and aurally. The segments were checked for marine mammal vocalizations and for what specific signals triggered each detector. The classification of marine mammal signals was based on the possible presence of the species in the area as described in various sources, and the specificity of the signals’ characteristics described in literature together with previous sound analysis experience (see further). All results presented here were manually confirmed.

Spectrograms for publication were created using Mathworks Matlab®.

#### Ambient sound levels

Broadband sound pressure levels (SPL), peak levels and third-octave band noise level measurements from the third-octave band centred at 25 Hz up to the band centred at 10079 Hz were calculated, which encompassed the frequencies corresponding to descriptors (D11.2) provided by the European Marine Strategy Framework Directive (2008/56/EC). Measurements were computed from shortly after deployment to before recovery of the recording equipment, therefore excluding operational self-noise, and moreover, the 1% highest pressure values were left out to reduce the influence of outlier events such as something knocking against the hydrophone.

## Electronic supplementary material

Graphical interface overview examples

## Data Availability

The datasets generated during and/or analyzed during the current study are available in the SONSETC repository, http://www.arctic.listentothedeep.com/.
