# Supplementary material for: Marine mammal acoustic detections in the Greenland and Barents Sea, 2013 – 2014 seasons
Source: Sci Rep. 2018 Nov 15;8:16882. doi: 10.1038/s41598-018-34624-z (PMC6237968; doi:10.1038/s41598-018-34624-z)
Supplement: Supplementary file 1 — Graphical interface overview examples [file 41598_2018_34624_MOESM1_ESM.pdf]

Marine mammal acoustic detections in the Greenland and Barents Sea, 2013 - 2014 seasons.

Steffen De Vreese[1], Mike van der Schaar[1], Jürgen Weissenberger[2], Florence Erbs[1], Monika Kosecka[1], Marta Solé[1], and Michel André[1*]

[1] Laboratory of Applied Bioacoustics, Technical University of Catalonia (UPC), BarcelonaTech, Rambla Exposición s/n, 08800 Vilanova i la Geltrú, Spain
[2] Statoil Norway
*michel.andre@upc.edu

Supplementary information (S1). Graphical interface overview.

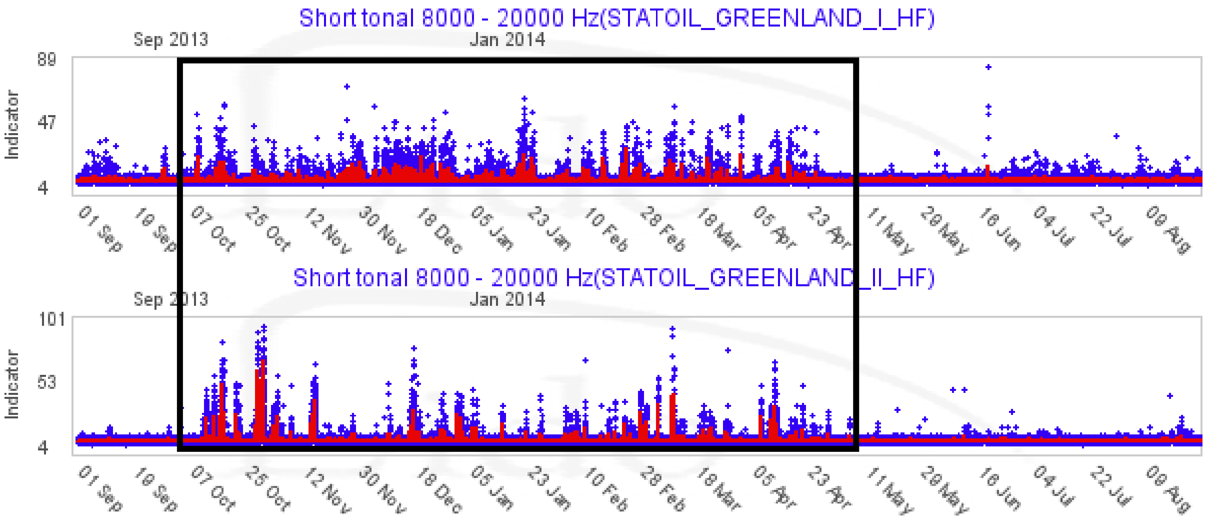

Figure 1. The output of the Short Tonal Detectors in the range of 8-20 kHz Hz in the Greenland stations. Sept 01, 2013 – Sept 1, 2014. Indications of the acoustic presence of probable bowhead whales. Each dot on the graph represents an audio segment. LINK.

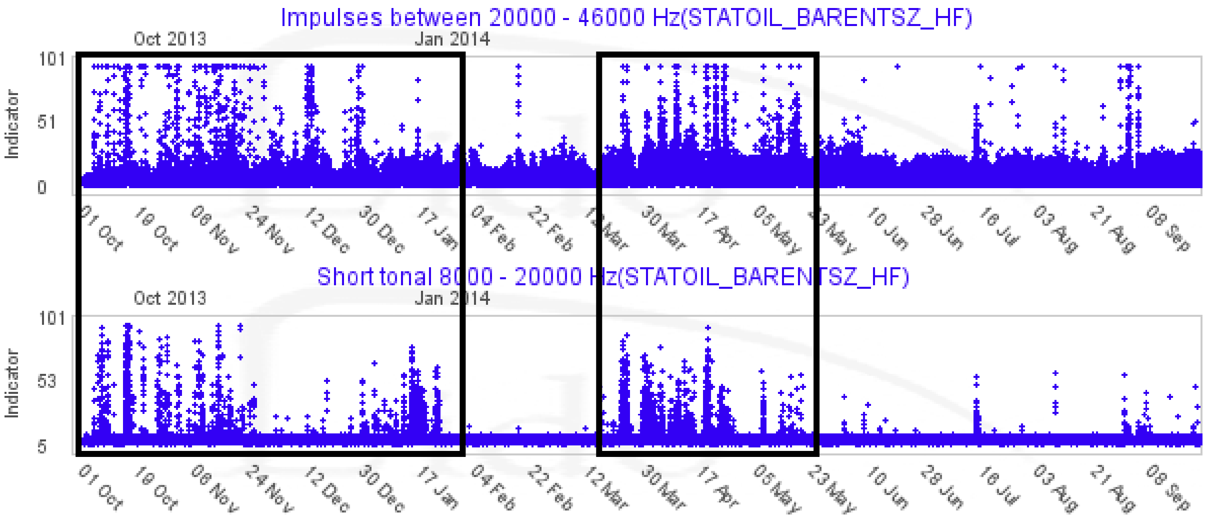

Figure 2. The output of a high-frequency impulse detector (20-60 kHz) and a short tonal detector (8-20 kHz) over the entire recording period in the Barents Sea. There are indications of the acoustic presence of delphinid species. LINK.

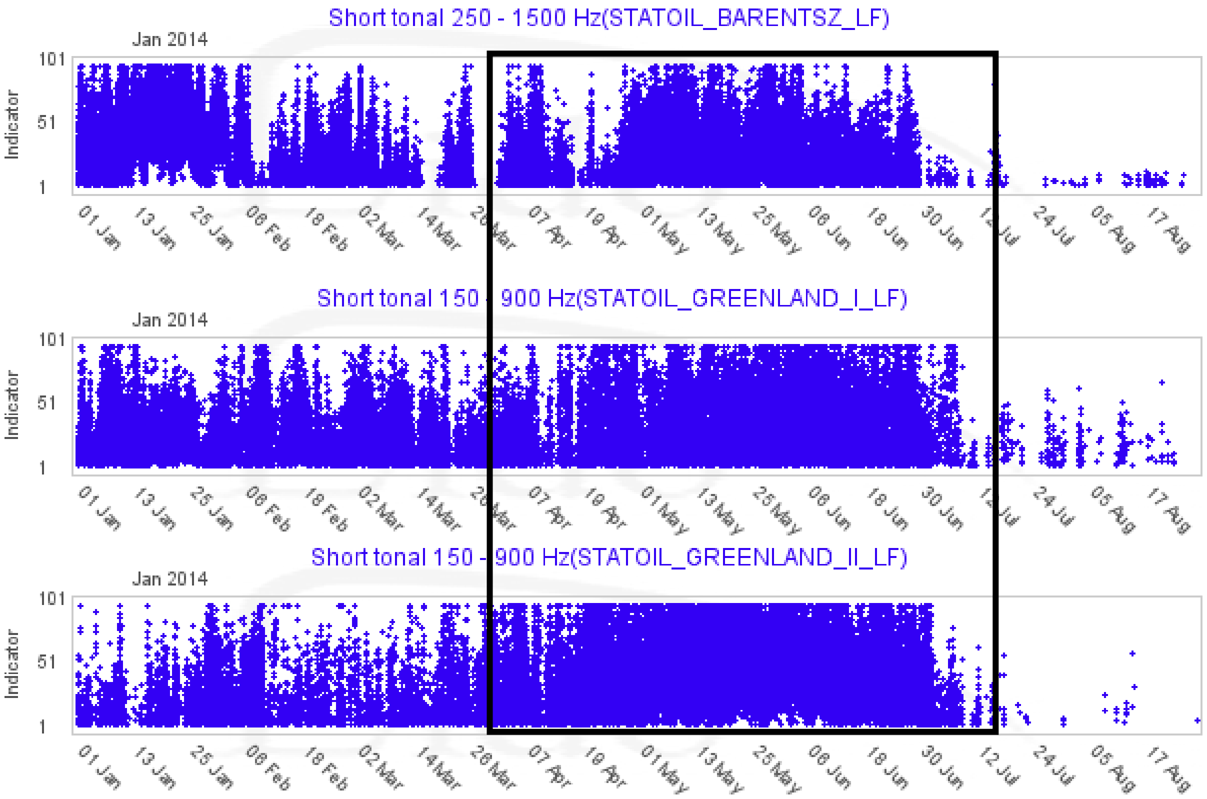

Figure 3. The output of the Short Tonal Detectors in the range of 150-1500 Hz in all three stations. Jan 1 – Oct 1. Indications of the acoustic presence of bearded seals. Each dot on the graph represents an audio segment. [LINK](#).
